# Takotsubo Syndrome: The First Non-Acute Proteomic Analysis by Remote Dried Blood Microsampling

**DOI:** 10.1007/s12265-026-10752-0

**Published:** 2026-02-27

**Authors:** Paul Marano, Kirstin Washington, Blandine Chazarin, Niveda Sundararaman, Koen Raedschelders, Jenna Maughan, Okezi Obrutu, Benita Tjoe, Romana Herscovici, Prizzi Moy, Chrisandra Shufelt, Thomas Rutledge, Alexander Polyak, Sandy Joung, Yunxian Liu, Susan Cheng, Janet Wei, Jennifer E. Van Eyk, C. Noel Bairey Merz

**Affiliations:** 1 Smidt Heart Institute, Cedars-Sinai Medical Center, Los Angeles, CA USA; 2 Barbra Streisand Women’s Heart Center, Cedars-Sinai Smidt Heart Institute, Cedars-Sinai Medical Center, 127 S. San Vicente Blvd, Los Angeles, CA 90048 USA; 3 Advanced Clinical Biosystems Research Institute, Cedars-Sinai Medical Center, Los Angeles, CA USA; 4 Department of Cardiac Surgery and Cardiology, Tel Aviv University, Tel Aviv, Israel; 5 Leviev Cardiothoracic and Vascular Center, Sheba Medical Center, affiliated to the Sackler School of Medicine, Tel Aviv University, Tel Aviv, Israel; 6 Mayo Clinic Women’s Health and Division of General Internal Medicine, Jacksonville, Florida, USA; 7 Psychology Service, VA San Diego Healthcare System, San Diego, California, USA

**Keywords:** Takotsubo syndrome, Proteomics, Stress cardiomyopathy

## Abstract

Takotsubo syndrome (TTS) is an under-recognized form of acute-onset heart failure typically precipitated by stress. While recovery of cardiac function is described over the course of weeks, adverse outcomes after apparent recovery are increasingly recognized. However, the pathophysiology of non-acute manifestations remains poorly understood. We used mass-spectrometry-based discovery proteomics from remotely collected non-acute dried blood microsamples to perform a case-control study in 62 participants with a prior TTS episode (median of 2.24 years prior to sample collection) and 47 reference controls. We quantified 398 unique proteins, and found that agnostic clustering techniques showed separation between TTS and reference control samples. This represents the first proteomic characterization of non-acute TTS. Pathway analysis of the 52 differentially regulated proteins demonstrated enrichment of proteins involved in complement activation, nitric oxide signaling, and with antioxidant activity. These enriched pathways may be suggestive of a persistent cardiomyopathy resulting from or predisposing to TTS.

**Graphical Abstract:**

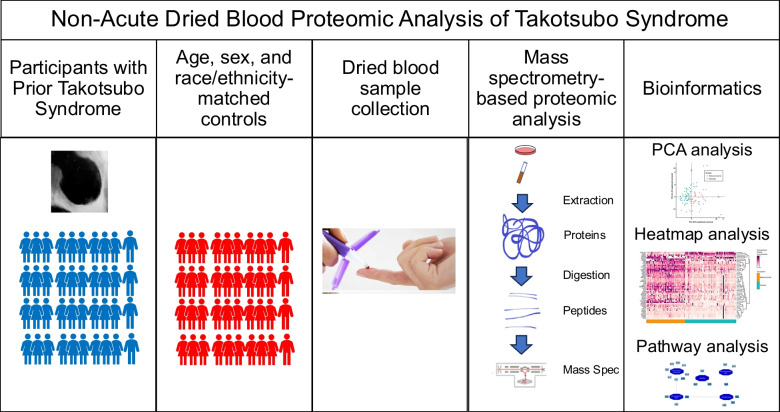

**Supplementary Information:**

The online version contains supplementary material available at 10.1007/s12265-026-10752-0.

## Introduction

Takotsubo syndrome (TTS) is an under-recognized form of acute-onset heart failure precipitated by intense emotional or physical stress, occurring predominantly in menopausal women [1]. The clinical presentation mimics a myocardial infarction, with chest pain and evidence of cardiac injury including electrocardiographic abnormalities, elevated circulating troponin, and left ventricular systolic dysfunction. Improved cardiac function is typical over the course of weeks, though mounting evidence indicates a majority of patients have persistent cardiac symptoms more than 1 year after the acute event [2], a subset develop recurrent TTS [3, 4], and 5-year mortality is comparable to patients with coronary artery disease [5, 6]. While limited plasma proteomic TTS studies have been conducted in the acute setting [7–9], there have not been proteomic investigations outside of the acute event, and the pathophysiology of the longer-term manifestations and therefore treatment strategies are unknown. To address these knowledge gaps, this study performed proteomics on dried blood samples from patients with prior TTS and reference controls.

## Methods

### Takotsubo Syndrome Population

The Cedars-Sinai Smidt Heart Institute Takotsubo Registry (NCT03910569) is an observational registry collecting retrospective and prospective data in TTS survivors [10]. Participants with a prior episode of TTS are recruited through patient-centered modalities including social media outreach and physician referral [10]. The registry has enrolled participants across 25 states in the United States and 3 additional countries (Canada, United Kingdom, Australia) [10]. Ethical approval for the registry was obtained by the Cedars-Sinai Medical Center Institutional Review Board. Detailed TTS medical records, including laboratory data, echocardiographic data, angiographic data and magnetic resonance imaging are retrospectively adjudicated according to InterTAK Diagnostic Criteria [1]. Participants provide demographic data and answer detailed physical and mental health questionnaires at baseline, which are then repeated annually [10]. Major adverse cardiovascular events and recurrent TTS events are adjudicated prospectively. At the time of registry enrollment, a dried blood sample is collected remotely. Consecutive participants with adjudicated TTS and available dried blood samples were included in the study.

### Reference Control Population

Reference control dried blood samples were collected by the same methodology from 47 age, sex, and race/ethnicity-matched participants in the Coronavirus Risk Associations and Longitudinal Evaluation (CORALE) study [11]. This cohort included a large, unselected population of 6,062 adults employed in a multi-site health care delivery system in Los Angeles County, of which a subset of 783 participants had dried blood samples collected. The study protocol was approved by the Cedars-Sinai Institutional Review Board.

### Dried Blood Sample Collection

Dried blood samples were collected via a 10-microliter volumetric absorptive blood microsampling device (Mitra device, Neoteryx) from a minimally invasive finger prick [12]. We have previously described the technique and feasibility of remote blood sample collection with this methodology in detail [13]. Briefly, the sample is obtained by a fingertip lance, blood is wicked up by the absorbent tip of the device, allowed to dry, and then placed in the provided Teflon bag with desiccant. The sample can then be mailed to the laboratory via standard mail, without the need for cold chain logistics.

### Sample Processing and Analysis

Once received by the laboratory, dried blood samples were stored at −80°C until analysis.

All samples selected were inspected and determined to be of good quality for sample processing. Samples were processed on Biomek i7 Automated Workstation (Beckman Coulter Life Sciences, Indianapolis, IN, US). Protein extraction and digestion were performed as previously reported [14]. Obtained peptide samples were decomplexified and quantified on a U3000 HPLC (Thermo) liquid chromatography coupled to Orbitrap Exploris 480 mass spectrometer (ThermoFisher Scientific). Data were acquired in data-independent acquisition mode using a 60-minute elution gradient, as previously described [12, 14]. Similarly, Bioreclamation blood samples were processed and analyzed at the same time as patient samples.

The robustness and the stability of the LC-MS platform was ensured by real-time monitoring based on iRT peptides added to all samples, using QuiC software (Biognosys). Also, independently digested Bioreclamation blood samples were repeatedly injected every 10 samples for an a posteriori validation of the reproducibility of the LC-MS platform. MS spectra were analyzed with OpenSWATH (v.1.0) using the Human Twin Plasma Library [15] with peptide and protein false discovery rate (FDR) set at 1%. Protein quantification was based on the intensity of proteotypic peptides only. Efficiency of the trypsin digestion was assessed by quantifying more than 80% of peptides without missing cleavage sites for all bioreclamation samples. Those samples also demonstrated more than 70% of identified peptides with a median coefficient of variation lower than 40%.

### Proteomics Data Analysis

Raw proteomic data were filtered, removing proteins that appeared in <50% of samples of either the TTS or reference control groups. Agnostic clustering used principal component analysis with the R package *FactoMineR.*

Statistical comparisons between TTS and reference control samples at the protein level were performed with mapDIA software, with pairwise comparisons using differential expression analysis based on a Bayesian latent variable model with Markov random field model [16]. Proteins were considered differentially expressed at the threshold of an absolute log2-fold change of 0.6 and a Bayesian FDR-adjusted *p* < 0.05.

Pathway analysis was performed to determine the canonical pathways for proteins that are differentially regulated in the TTS group compared with the reference control group using Protein Interaction Network Extractor (PINE), an automated graphical user interface application for visualization and exploration of global proteome networks [17]. The Cytoscape App, ClueGO [18], was applied to perform functional enrichment analysis that searches across multiple public databases including Gene Ontology (GO) [19], and as Kyoto Encyclopedia of Genes and Genomes (KEGG) [20], Reactome [21], and WikiPathways [22]. Pathways were considered significantly enriched at a threshold of a Bonferroni-corrected p-value < 0.05.

For further data interpretation, the whole quantified proteome was explored and mapped to pathways of interest based on observations from previously published preclinical data and clinical descriptions. To highlight protein regulations involved in nitric oxide-related pathways, individual intensity values for each protein with the annotation “nitric oxide binding” (2 proteins, GO:0070026) and “positive regulation of nitric oxide biosynthetic process” (6 proteins, GO:0045429), from Gene Ontology database, were represented on a heatmap. In the same way, protein intensities were compiled on a heatmap for the proteins involved in “complement and coagulation cascade” pathway from KEGG pathway database (37 proteins, hsa04610).

We also performed multivariable linear regression to evaluate for associations between several predictor variables and the protein expression level of the 5 top differentially expressed proteins (i.e. the proteins with the largest fold change in comparison between Takotsubo participants and reference controls). The following predictor variables were included in the model: history of TTS, age, history of diabetes, hypertension and malignancy, and history of medication use including ACEi/ARB, *β*-blocker, statin and aspirin use. A coefficient score is reported that measures the strength and direction of linear relationship between each predictor variable and the protein expression level while accounting for other variables in the model, along with a p-value that determines statistical significance of the relationship.

### Data Availability

The majority of the data presented in this article are provided in the figures and tables. The detailed methods used for proteomic analysis are available from the Advanced Clinical Biosystems Research Institute at Cedars-Sinai Medical Center. The raw proteomics data coupled to de-identified participant data will be provided on reasonable request.

## Results

### Clinical Characteristics of Groups

Clinical characteristics of the TTS (*n*=62) and reference control groups (*n*=47) are provided in Table 1. The 62 consecutive adjudicated TTS participants with available dried blood samples were all women, with median age 62.5 years (interquartile range 58.0–70.0.0.0), 95% non-Hispanic White, 42% with a history of hypertension, 11% with a history of diabetes, 13% with a history of malignancy, and 33% with a history of depression or anxiety. The age-, sex- and race/ethnicity-matched reference controls had a median age of 59.0 years (interquartile range 49.5–61.0.5.0), 25% had a history of hypertension, 3% had a history of diabetes, 18% had a history of malignancy, and 28% had a history of depression or anxiety. There was a higher rate of selected medication use in the TTS participants compared to the reference controls at the time of sample collection, including angiotensin-converting enzyme inhibitor (ACEi) or angiotensin II receptor blocker (ARB), *β*-Blocker, statin, and aspirin (Table 1).

**Table 1:** Clinical characteristics of reference controls and participants with prior Takotsubo syndrome

Variable	Reference Controls (*n*=47)*	Takotsubo syndrome (*n*=62)
Age at sample collection, y	59.0 [49.5–61.0]	62.5 [58.0–70.0]
Female Sex, n (%)	47 (100%)	62 (100%)
Self-identified White Race, n (%)	45 (96%)	60 (97%)
Self-identified Hispanic Ethnicity, n (%)	4 (9%)	3 (5%)
Clinical Features		
Body mass index	25.8 ± 5.0	25.4 ± 5.7
Hypertension, n (%)	10 (25%)	26 (42%)
Diabetes, n (%)	1 (3%)	7 (11%)
Dyslipidemia, n (%)	23 (58%)	25 (41%)
Malignancy, n (%)	7 (18%)	8 (13%)
Depression/Anxiety, n (%)	11 (28%)	20 (33%)
Chronic kidney disease, n (%)	0 (0%)	1 (2%)
Chronic obstructive pulmonary disease, n (%)	3 (8%)	7 (12%)
Thyroid Disease, n (%)	13 (33%)	15 (25%)
Medications, n (%)		
ACEi/ARB, n (%)	6 (15%)	27 (44%)
β-Blocker, n (%)	5 (13%)	36 (58%)
Statin, n (%)	9 (23%)	26 (42%)
Aspirin, n (%)	4 (10%)	28 (45%)
P2Y12i, n (%)	0 (0%)	4 (6%)
Inhaled beta-agonist, n (%)	3 (8%)	3 (5%)
Antidepressant, n (%)	4 (10%)	13 (21%)

Data are presented as median [25^th^−75^th^ percentile] or *n* (%)

*Data on age, sex, race, and ethnicity are available for all 47 reference controls. Data on the additional clinical characteristics and medication use were available for 40 of the 47 reference controls

*ACEi* indicates angiotensin-converting enzyme inhibitor, *ARB* angiotensin II receptor blocker, *P2Y12i*, P2Y12 receptor inhibitors

Dried blood samples were collected at study enrollment for each participant with prior TTS, with a variable time between TTS event and sample collection (median of 2.24 years after most recent TTS event, interquartile range 0.55–4.82). Details about the prior TTS events are included in Table 2. There were 11 participants (18%) with a history of recurrent TTS, while the remaining participants had a single TTS event. Echocardiography demonstrated recovery of left ventricular function in all patients.

**Table 2:** Clinical details of the prior takotsubo syndrome episodes

Variable	Takotsubo syndrome (*n*=62)
Age at first adjudicated TTS event, y	60 [54–67]
Participants with recurrent TTS events	11 (18%)
Total number of TTS events	79
Time from most recent adjudicated TTS event to sample collection, y	2.24 [0.55–4.82]
Triggering Factors for TTS event	
Emotional trigger only	27 (34%)
Physical trigger only	15 (19%)
Both emotional and physical trigger	13 (16%)
None identified	23 (29%)
ST-segment elevation at presentation	13 (16%)
Maximum troponin during TTS event	0.68 [0.24–3.03]
Left ventricular ejection fraction at presentation, %	37.6 ± 12.8
Wall motion type	
Apical	43 (54%)
Mid-ventricular	8 (10%)
Basal	6 (8%)
Left ventricular ejection fraction at recovery, %	60.4 ± 6.1
In-hospital complications	
Cardiogenic shock	5 (6%)
Invasive mechanical ventilation	4 (5%)
Non-invasive mechanical ventilation	3 (4%)
Atrial fibrillation/flutter	2 (3%)
Ventricular tachycardia	1 (1%)

Data are presented as mean ± standard deviation for normally distributed variables, median [25^th^−75^th^ percentile] or n (%).

*TTS* indicates Takotsubo syndrome.

### Principal Component Analysis Demonstrates Agnostic Clustering

**Fig. 1:**
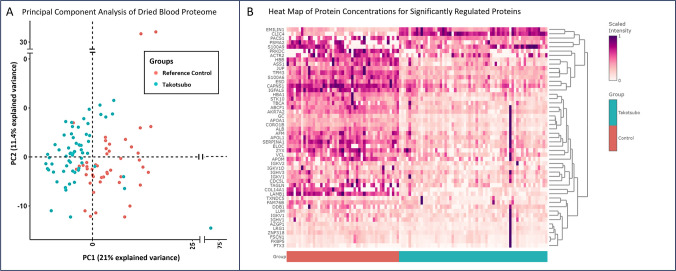
(**A**) Principal Component Analysis of Dried Blood Proteome. Unsupervised principal component analysis (PCA) including all 398 quantified proteins shows that principal components 1 and 2 account for 21.0% and 11.4% of the variance in protein concentrations, respectively. Annotation of the samples as TTS samples (teal circles, *n*=62) and reference control samples (orange circles, *n*=47) demonstrates distinct clustering. (**B**) Heatmap of Protein Concentrations for Significantly Regulated Proteins. Heatmap showing the blood protein concentration for each of the 52 differentially expressed proteins (each row represents a differentially expressed protein, indicated by the corresponding gene names) in all participants (each column represents a single participant). TTS participants are represented with the teal annotation (“Takotsubo"), and reference controls with the orange annotation (“Reference Control”). Proteins were considered differentially expressed at the threshold of an absolute log2-fold change of 0.6 and a false discovery rate-adjusted *p* < 0.05. EMILIN1 – EMILIN-1, CLIC4 - Chloride intracellular channel protein 4, PACS1 - Phosphofurin acidic cluster sorting protein 1, PSMA2 - Proteasome subunit alpha type-2, S100A9 - Protein S100-A9, PRKDC - DNA-dependent protein kinase catalytic subunit, ACTR2 - Actin-related protein 2, HBB - Hemoglobin subunit beta, ASS1 - Argininosuccinate synthase, JUP - Junction plakoglobin, TPM3 - Tropomyosin alpha-3 chain, S100A6 - Protein S100-A6, ESD - S-formylglutathione hydrolase, CAPNS1 - Calpain small subunit 1, IGFALS - Insulin-like growth factor-binding protein complex acid labile subunit, HBA1 - Hemoglobin subunit alpha, STK10 - Serine/threonine-protein kinase 10, TBCA - Tubulin-specific chaperone A, ABCF1 - ATP-binding cassette sub-family F member 1, AKR7A2 - Aflatoxin B1 aldehyde reductase member 2, GC - Vitamin D-binding protein, APOA1 - Apolipoprotein A-I, CORO1B - Coronin-1B, ALB - Albumin, AFM - Afamin, APOL1 - Apolipoprotein L1, SERPINA1 – Alpha-1 antitrypsin, ELOC - Elongin-C, ZYX - Zyxin, VCL - Vinculin, APOM - Apolipoprotein M, IGKV2 - Immunoglobulin kappa variable 2–30, IGKV1D - Immunoglobulin kappa variable 1D-16, IGHV3 - Immunoglobulin heavy variable 3–23, IGKV1 - Immunoglobulin kappa variable 1–39, CDC5L - Cell division cycle 5-like protein, TAGLN - Transgelin-2, COL14A1 - Collagen alpha-1(XIV) chain, LAMB1 - Laminin subunit beta-1, TXNDC5 - Thioredoxin domain-containing protein 5, FAM76B - Protein FAM76B, DDB1 - DNA damage-binding protein 1, LUM - Lumican, IGKV1 - Immunoglobulin kappa variable 1–33, IGHV1 - Immunoglobulin heavy variable 1–2, AZGP1 - Zinc-alpha-2-glycoprotein, LRG1 - Leucine-rich alpha-2-glycoprotein, ZNF318 - Zinc finger protein 318, FSCN1 - Fascin, FKBP5 - Peptidyl-prolyl cis-trans isomerase FKBP1A, PTX3 - Pentraxin-related protein

A total of 398 unique proteins were quantified from the dried blood samples. Using the whole quantified proteome, unsupervised principal component analysis (PCA) demonstrated a clustering of samples into two distinct groups with separation between TTS and reference control samples (Fig. 1A). Principal component 1 accounted for 21.0% of the variance, and principal component 2 accounted for 11.4% of the variance between samples.

Among TTS samples, there was no distinct separation in PCA between participants for whom dried blood samples were obtained recently after TTS event (< 1 year) or more remotely, either between 1 and 5 years, or greater than 5 years after their TTS event (Supplemental Figure 1A). Similarly, there was no distinct separation between TTS participants aged less than 60 years, between 60 and 70 years, or greater than 70 years (Supplemental Figure 1B). Furthermore, PCA clustering did not demonstrate a separation between TTS participants under or without medical treatment including angiotensin converting enzyme inhibitors or angiotensin receptor blockers, *β*-blockers, statins, or aspirin (Supplemental Figure 2). *Differentially regulated proteins and pathway analysis*

Out of the 398 quantified unique proteins, 52 proteins were significantly regulated (log2-fold change of > |0.6|, FDR-adjusted p-value ≤0.05) in the TTS group compared to the reference control group (Fig. 1B). On further analysis with multivariable linear regression, the history of TTS was a statistically significant predictor of the protein expression level for all of the top 5 differentially expressed proteins, after adjusting for the effect of other predictor variables including age, history of diabetes, hypertension and malignancy, and history of medication use including ACEi/ARB, *β*-blocker, statin and aspirin use (Supplemental Table, A-E).

Pathway analysis performed on the 52 differentially regulated proteins using PINE with the GO and KEGG databases highlighted significant enrichment of biological pathways, including “complement activation, classical pathway,” “nitric oxide biosynthetic process,” and “antioxidant activity.” (Table 3).

**Table 3:** Selected pathways that are differentially regulated between participants with prior Takotsubo syndrome and reference controls

Pathway Description	No. of Differentially Regulated Proteins	Total No. of Proteins in Pathway	Bonferroni-corrected p value
Complement activation, classical pathway	8	43	4.38 x 10^−7^
Regulation of complement activation	7	45	1.33 x 10^−6^
Nitric oxide biosynthetic process	4	5	5.04 x 10^−4^
Positive regulation of nitric oxide biosynthetic process	4	46	8.49 x 10^−5^
Stress fiber	3	110	0.0011
Lamellipodium organization	4	53	6.75 x 10^−4^
Cellular detoxification	6	325	2.79 x 10^−5^
Antioxidant activity	5	173	1.40 x 10^−4^
High-density lipoprotein particle	4	55	2.35 x 10^−5^
High-density lipoprotein particle remodeling	3	29	1.90 x 10^−4^

### Complement Proteins are Differentially Regulated in Prior TTS

**Fig. 2:**
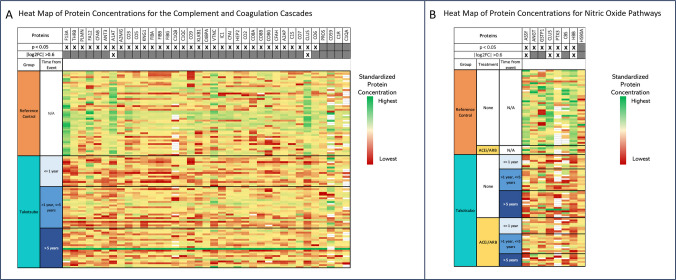
(**A**) Heat Map of Protein Concentrations in the Complement and Coagulation Cascades. There were 37 total proteins from the “complement and coagulation cascade” pathway of the Kyoto Encyclopedia of Genes and Genomes database identified in our analysis. Of these, 33 were differentially regulated based on a false discovery rate-adjusted p-value < 0.05, allowing for any fold change. There were 2 proteins that were differentially regulated by the criteria of an absolute log2-fold change of 0.6 and a false discovery rate-adjusted *p* < 0.05: clusterin (CLUS) and alpha-1 antitrypsin (A1AT). A heat map is shown depicting relative protein concentrations. Each row represents a single participant, and each column represents a protein within the enriched pathways (indicated by the corresponding gene name). The log2 fold change and the false-discovery rate-adjusted p-value are indicated in the first two rows. TTS participants are grouped based on whether the dried blood samples were obtained < 1 year after TTS event, between 1 and 5 years after the TTS event, or greater than 5 years after their TTS event. The differential expression between TTS participants and reference controls is visually preserved across these subgroups of TTS participants. F13A – Coagulation factor XIIIA, THRB – Prothrombin, PLMN – Plasminogen, FA12 – Coagulation Factor XII, CFAB – Complement Factor B, ANT3 – Antithrombin 3, A1AT – Alpha-1 antitrypsin, A2MG – Alpha-2 macroglobulin, CO3 – Complement C3, CO5 – Complement C5, KNG1 – Kininogen-1, FIBA – Fibrinogen alpha chain, FIBB – Fibrinogen beta chain, C1QB – Complement C1q subcomponent subunit B, C1QC - Complement C1q subcomponent subunit C, CO9 – Complement C9, KLKB1 - Kallikrein, C4BPA – C4b-binding protein alpha chain, VTNC - Vitronectin, IC1 – Plasma protease C1 inhibitor (SerpinG1), CFAI – Complement factor I, HEP2 – Heparin cofactor 2, CO2 – Complement C2, CO8A – Complement C8 alpha chain, CO8B – Complement C8 beta chain, CO8G – Complement C8 gamma chain, CFAH – Complement factor H, A2AP – Alpha-2 antiplasmin, C1S – Complement C1s subcomponent, CO7 – Complement C7, CLUST - Clusterin, CO6 – Complement C6, PROS – Vitamin K-dependent protein S, CD59 – CD59 glycoprotein, C1R – Complement C1r subcomponent, C1QA - Complement C1q subcomponent subunit A. (**B**) Heat Map of Protein Concentrations for Nitric Oxide Pathways. Based on pathway analysis, the Gene Ontology Biological Process pathway “positive regulation of nitric oxide biosynthetic process” was enriched for differential regulation between TTS and Reference Control samples. There were 8 proteins in the pathways “nitric oxide binding” and “positive regulation of nitric oxide biosynthetic process” identified in the samples, of which 4 were differentially regulated by the criteria of an absolute log2-fold change of 0.6 and a false discovery rate-adjusted *p* < 0.05. A heat map is shown depicting standardized protein concentrations. Each row represents a single participant, and each column represents a protein within the enriched pathways (indicated by the corresponding gene name). The log2 fold change and the false-discovery rate-adjusted p-value are indicated in the first two rows. The TTS participants were further divided into whether or not they were treated with angiotensin-converting enzyme inhibitor (ACEi) or angiotensin II receptor blocker (ARB), medications known to impact nitric oxide. The TTS participants were also divided based on whether the dried blood samples were obtained < 1 year after TTS event, between 1 and 5 years after the TTS event, or greater than 5 years after their TTS event. Visually, there was no pattern of differential protein expression between TTS participants in these subgroups. ASSY – Argininosuccinate synthase, ANGT - Angiotensinogen, GSTP1 – Glutathione S-transferase, CLUS - Clusterin, PTX3 – Pentraxin-related protein, CBS – Cystathionine beta-synthase, HBB – Hemoglobin subunit beta, HS90A – Heat shock protein HSO 90-alpha

Given the enrichment of multiple pathways related to complement activation, proteins in the complement and coagulation cascades were further analyzed. There were 37 proteins in the complement and coagulation cascades identified in the analysis, of which 33 were differentially regulated based on FDR-adjusted p-value ≤ 5%, with any fold change (Fig. 2A). The proteins clusterin (CLUS) and alpha-1 antitrypsin (A1AT) were differentially regulated by the criteria of an absolute log2-fold change of 0.6 and a FDR-adjusted *p* < 0.05 (Fig. 2A). In order to evaluate the potential impact of the remoteness of the TTS event on the regulation of proteins in this pathway, TTS participants were separated into different groups based on whether the dried blood samples were obtained recently after TTS event (< 1 year) or more remotely, either between 1 and 5 years, or greater than 5 years after their TTS event. The differential regulation of proteins in this pathway compared to reference controls was preserved across these different TTS groups (Fig. 2A).

### Nitric Oxide Signaling Proteins are Differentially Regulated in Prior TTS

Proteins related to nitric oxide signaling were further analyzed (Fig. 2B) due to the enrichment of nitric oxide-related pathways. Out of the 5 total proteins with the annotation “nitric oxide binding,” 2 were quantified and differentially regulated in our study, and 6 proteins with the annotation “positive regulation of nitric oxide biosynthesis” (out of the 46 possible proteins with this annotation) were quantified and differentially regulated in our study. There was downregulation in 7 of these 8 proteins (Fig. 2B). Separation of the TTS patients into distinct groups based on the time from the TTS event to sample collection (< 1 year, 1 to 5 years, > 5 years) showed that this downregulation was preserved across each group. Further, separation into groups based on the use of medications that might impact nitric oxide signaling (ACEi/ARB), showed that the difference between TTS and reference control samples was preserved, irrespective of medication use (Fig. 2B).

### Extracellular Matrix Proteins are Differentially Regulated in Prior TTS

In addition to the pathway enrichment analysis, manual review of the differentially expressed proteins revealed that 10 of the 52 differentially regulated proteins shared the annotation of the GO cellular component term “collagen-containing extracellular matrix” (out of 1,282 possible proteins with this annotation). The proteins collagen alpha-1(XIV), lumican and elastin microfiber interfacer 1 were among the proteins that were differentially expressed in TTS samples compared to reference control samples.

## Discussion

This study is the first to characterize the blood proteome for non-acute TTS (median of 2.24 years after the TTS event), highlighting a proteomic signature distinct from reference controls. Despite the previous conception of TTS as a transient condition, this study contributes to the growing body of evidence demonstrating persistent abnormalities in patients with a prior episode of TTS. Registry studies showed that after a TTS event, there is a risk of recurrent TTS and mortality is elevated above the general population [5, 6]. Recently, a study focused on patients who experienced a TTS episode more than 1 year prior demonstrated objective evidence of persistent cardiac structural and functional abnormalities, including prolonged native T1 times on cardiac magnetic resonance, which may suggest microscopic fibrosis [2]. Previous efforts to identify circulating biomarkers that distinguished patients with prior Takotsubo from reference controls have not been fruitful [2], due in part to a lack of pathophysiologic understanding of long-term manifestations.

We used discovery proteomics with mass spectrometry-based analysis to identify blood proteins that are differentially regulated in patients with prior TTS event(s). Proteomic profiling has demonstrated efficiency in identifying biomarkers and providing insights into pathophysiology in other cardiac conditions including hypertrophic cardiomyopathy, dilated cardiomyopathy, and pulmonary arterial hypertension [23–25]. With this approach, we identified a distinct blood proteome in participants with prior TTS compared to reference controls and propose multiple contributory biologic pathways. These findings lay an important groundwork to understand the biologic pathways that are differentially regulated after the acute TTS event, toward the aims of developing tests for risk stratification for patients with prior TTS and identifying targets for treatment.

Our study has several important strengths. We used data-independent-acquisition MS, a next-generation proteomics technique with excellent reproducibility and high accuracy for quantitation across a wide range of protein abundance [26]. We purposefully studied patients remote from their most recent TTS event, to ensure that the protein profile we investigated was reflective of long-term changes related to the acute TTS event and is therefore novel. In addition, we used a dried blood matrix collected remotely from a fingerprick, a proof-of-concept that this sampling technique can be used for discovery proteomics in cardiovascular diseases [27]. Through regression analysis, we also demonstrated that TTS is a predictor of the protein changes we observed even after adjusting for differences in clinical characteristics and medication use between groups.

### Pathway Analysis of Circulating Proteins Suggests a Persistent Cardiomyopathy

The pathways that were significantly enriched for differentially expressed proteins between TTS samples and reference controls provide a starting point for understanding the processes involved in non-acute TTS. Among the pathways we identified by this analysis and by manual review were the “collagen-containing extracellular matrix” and “complement activation, classical pathway.” Similarly, proteins involved in extracellular matrix remodeling and inflammation, including the complement and coagulation systems, have been previously shown to be associated with incident heart failure [28, 29]. While we cannot determine whether the origin of these circulating proteins is cardiac, the alignment with previous heart failure proteomic studies is suggestive of an ongoing cardiomyopathy in non-acute TTS.

### Extracellular Matrix Proteins – Potential Circulating Biomarkers of Cardiac Fibrosis

We found multiple proteins in the “collagen-containing extracellular matrix” pathway were differentially regulated in TTS blood samples vs reference controls, including the proteins collagen alpha-1(XIV), and lumican. There is precedent for the importance of circulating extracellular matrix proteins in TTS; in a prior study of plasma samples during the acute phase of TTS, the pathway “extracellular matrix-receptor interaction” was significantly enriched in acute TTS compared to both acute myocardial infarction patients and compared to normal controls [7].

While the source of these circulating extracellular matrix proteins is not clear from our study, as these proteins are expressed ubiquitously, there is a growing body of evidence that circulating proteins can be associated with extracellular changes in the heart [30, 31]. A recent plasma proteomic investigation in a community cohort found several proteins associated with cardiac magnetic resonance imaging indicators of fibrosis [32]. In addition, prior work in heart failure patients has established circulating biomarkers, all related to collagen, to be associated with histologically proven myocardial interstitial fibrosis [31]. While these previously investigated biomarkers were not identified with our methodology, the related proteins we identified in the “collagen-containing extracellular matrix” are candidates for further exploration based on our data and preclinical studies.

The largest fold change we observed between TTS and reference control samples was in the protein collagen alpha-1(XIV), which was downregulated in TTS. This protein has a role in regulating the formation and organization of collagen fibrils in connective tissue [33]. In a mouse knockout model, collagen XIV deficiency led to a greater increase in LV wall thickening in response to pathological pressure overload compared to wild-type animals [34]. Similarly, lumican, another extracellular matrix protein that regulates collagen fibril assembly, was downregulated in our TTS samples. Lumican knockout mice responded adversely to stress with isoproterenol administration, demonstrating increased fibrosis, worse left ventricular function and increased death compared to wild-type mice [35]. While our study only assessed the circulating levels of proteins, which may not reflect low concentrations in cardiac tissue as would be seen in a genetic knockout animal model, it is possible that the lower circulating concentrations of these proteins indicate an underlying susceptibility to adverse remodeling in response to an inciting stress. Future studies will be important to validate the differential regulation of these extracellular matrix proteins in a larger cohort including through the use alternative methodology for protein quantification, and to correlate circulating protein changes with cardiac imaging to confirm their association with TTS-related cardiac remodeling.

### Dysregulation of the Complement Pathway may Represent Predisposition Toward Inflammation

We observed differential regulation of multiple proteins involved in complement activation. This finding is of particular interest, as previous work supports an acute inflammatory picture during the acute TTS event, though it remains unclear if acute inflammation is a cause or a consequence of the acute event [36]. A previous plasma proteomic evaluation during the acute event showed significant enrichment in the complement cascade in TTS patients compared with normal controls, including significant downregulation of C3 and C4, reflecting activation of the classical complement pathway [7]. Our data in participants with remote prior Takotsubo syndrome also demonstrates activation of the classical complement pathway, with downregulation of C3 (Fig. 2A).

Prior reports have used advanced imaging techniques and histology of post-mortem cardiac tissue to demonstrate a macrophage-predominant inflammatory infiltrate in the myocardium acutely [36, 37]. In addition, prior work has identified elevated circulating levels of plasma cytokines including IL-6, both acutely and at 5 months after the TTS event, suggesting systemic inflammation [36]. While IL-6 was not among the proteins we quantified with our assay, our data with persistent differential regulation of complement proteins lends further support to the presence of an ongoing pro-inflammatory state, long after a TTS event.

Notable observations in our study include the downregulation in TTS samples of clusterin and SerpinG1 proteins, both of which inhibit complement-mediated inflammation (Fig. 2A). Clusterin, also known as apolipoprotein J, is a ubiquitous glycoprotein with multiple reported functions, including inhibition of complement-mediated cytolysis [38]. During an acute myocardial infarction, prior studies have demonstrated an acute decrease in plasma clusterin levels and increased myocardial cell concentration of clusterin in the infarction and peri-infarction zone [39, 40]. In a preclinical model of myocardial infarction, administration of clusterin decreased infarction size [41]. Further, low plasma levels of clusterin have been associated with adverse prognosis in heart failure patients [42]. This prior work has led to speculation that circulating clusterin is consumed by inflammation in chronic heart failure [42], which represents an interesting potential hypothesis to explain our findings of downregulated clusterin in patients with prior TTS.

SerpinG1 is also downregulated in TTS samples in our study. SerpinG1, also known as C1-inhibitor, is an anti-inflammatory protein that inhibits activation of the complement cascade (Fig. 2A) [43]. It has been described to be upregulated in the left ventricular myocardium in proteomic analysis of ischemic cardiomyopathy [44], while a low plasma SerpinG1 level was part of a 4 protein score to predict mortality in cardiogenic shock [45]. Downregulation of SerpinG1 in TTS samples may similarly identify a systemic inflammatory state. These observations together may represent an underlying predisposition to overactivation of inflammatory pathways in the presence of an acute trigger or may be sequelae of the prior acute event.

Another protein of interest is Apolipoprotein M, which is downregulated in TTS samples. Apolipoprotein M is a chaperone protein for a sphingolipid that activates G-protein–coupled receptors and the phosphoinositide 3-kinase signaling pathway. Animal studies suggested that Apolipoprotein M promotes anti-inflammatory effects, survival of cardiomyocytes, and improved endothelial function [46]. Reduced level of Apolipoprotein M has been associated with adverse outcomes in heart failure [47, 48]. In our study, the downregulation of apolipoprotein M in blood may support the notion that patients with prior TTS have a chronic heart failure phenotype.

### Downregulation of Nitric Oxide Synthesis and Antioxidant Proteins

Our analysis also demonstrated differential regulation in TTS samples of multiple proteins related to nitric oxide synthesis and antioxidant activity. Previously, two studies have suggested abnormalities in nitric oxide signaling in patients with remote prior TTS; when subjected to a cold pressor test or mental stress, these patients demonstrated abnormal vasoconstrictor responses, suggesting persistent endothelial dysfunction [49, 50]. Consistent with those findings, we observed a downregulation of multiple proteins that positively regulate nitric oxide biosynthesis, including argininosuccinate synthetase-1, the rate-limiting enzyme in nitric oxide production [51].

Multiple studies in humans and animal models support a role for nitrosative and oxidative stress as mediators of the inflammatory response and myocardial injury that occur after the initial catecholamine surge [52, 53]. Our work offers further support for the importance of these pathways in TTS. We observed downregulation in multiple proteins with antioxidant activity, including cystathionine beta-synthase in TTS compared to reference controls. This protein is important for its role in the production of hydrogen disulfide, a scavenger of reactive oxygen species that provides a protection against oxidative stress. Prior animal model research has demonstrated decreased plasma and myocardial hydrogen sulfide levels in TTS and showed that treatment with sodium hydrosulfide recovers hydrogen sulfide levels and improves outcomes [54].

## Study Limitations

We cannot discern whether changes in the proteome that we observe preceded the acute TTS event and represent a predisposition to TTS, or represent sequelae of the acute event. We have proteomic data for a single time point for each patient, and these time points vary with respect to the length of time after the acute TTS event. Our analysis evaluating for agnostic clustering based on the time from event to sample collection (Supplemental Figure 1A) and looking at protein quantification stratified by time in pathways of interest (Fig. 2), provides reassurance that the changes we observed are durable. Future longitudinal studies, tracking these protein changes over time including before and after recurrent TTS events, will be helpful in distinguishing whether these changes predispose to TTS or occur as a result of the TTS event.

We acknowledge a limited sample size in this initial exploratory investigation of the proteomics of TTS outside of the acute event. One important challenge of this study is the potential impact of factors other than the history of TTS on the blood proteome. Prior work has shown that a substantial amount of variation in potential cardiovascular biomarkers can be attributed to non-disease factors, such as genetics and lifestyle [55, 56]. While matching for key variables between TTS participants and reference controls is a strength of our study, we recognize that differences between the groups may impact the proteome, including between-group differences in rates of hypertension and diabetes, and differential treatment with medications including ACEi/ARB, *β*-blockers, statins and antiplatelet agents. We therefore conducted additional analyses to evaluate the impact of multiple variables on the plasma proteome.

We used principal component analysis to look for agnostic clustering based on medication use (Supplemental Figure 2). The lack of distinct clustering based on these variables strengthens the evidence that the history of TTS is an important driver of the differentially regulated proteins we observed. Further, within biologic pathways of interest, we observed that the differential regulation of the majority of proteins in TTS compared to reference control was preserved with or without the presence of these medications (Fig. 2B). We also performed multivariable linear regression (Supplemental Table), confirming that the history of TTS was a significant predictor of the protein expression level of the top 5 differentially expressed proteins even after adjustment for age, clinical factors including diabetes, hypertension and malignancy, and the use of medications including ACEi/ARB, *β*-blockers, statins and aspirin. Ultimately, larger studies and comparisons between TTS patients and patients with other cardiac diseases, including those with a prior myocardial infarction, will be necessary to isolate the impact of prior TTS on the proteome.

While the use of a dried blood matrix for discovery proteomics is notable and has the potential to increase accessibility in clinical trials and in precision medicine, we note that the number of quantified proteins (398) is lower than what has been reported for prior proteomic studies in cardiovascular disease using mass spectrometry on plasma samples or using aptamer technology [7, 23, 45, 58]. The variability in the quantified proteome can be associated with multiple factors, including different instruments, different sample collection methods and processing techniques, as well as different matrices (dried blood vs plasma) [27]. In comparison with aptamer technology, however, we note that our data-independent acquisition mass spectrometry is not limited by a pre-selected set of candidate proteins.

Additionally, while the inclusion of only female participants could be perceived as a limitation, we note that about 90% of TTS patients are women according to registry data, providing an important rationale for our design [6]. Further study is needed to evaluate the external validity of these findings in men and in other racial groups.

## Conclusions

These results demonstrate, for the first time, non-acute TTS blood proteome characterization at a median of 2.24 years after the most recent TTS event, showing a proteomic signature distinct from that of reference controls. Our data provide evidence for chronic dysregulation of the complement proteins, proteins associated with nitric oxide synthesis and antioxidant activity, as well as select proteins related to the collagen-containing extracellular matrix. In regression analysis, the history of TTS was a significant predictor of protein expression levels even after adjustment for clinical characteristics and medication use. While these results cannot differentiate a pre-disposition to acute TTS versus longer-term sequelae of TTS injury, these proteomic signatures can generate hypotheses for future dedicated investigations into the long-term pathophysiology of TTS.

## Supplementary Information

ESM 1 (DOCX 360 kb)

## Abbreviations

ACEi: angiotensin converting enzyme inhibitor
ARB: angiotensin II receptor blocker
FDR: false discovery rate
GO: Gene Ontology
KEGG: Kyoto Encyclopedia of Genes and Genomes
LC-MS: liquid chromatography-mass spectrometry
MS: mass spectrometry
PINE: Protein Interaction Network Extractor
PCA: Principal component analysis
TTS: Takotsubo syndrome

## Declarations

### Clinical Relevance

Although improvement in cardiac function is typical over the course of weeks after a Takotsubo syndrome event, adverse outcomes after apparent recovery are now recognized. The pathophysiology of these longer-term manifestations remains poorly defined. We demonstrate a distinct blood proteome in Takotsubo syndrome survivors compared with reference controls. This work supports a chronic heart failure phenotype after a Takotsubo syndrome event, and underscores the importance of long-term monitoring and treatment of these patients.

### Clinical Trial Registration

NCT03910569.

## References

[1] Ghadri J-R, Wittstein IS, Prasad A, Sharkey S, Dote K, Akashi YJ, et al. International Expert Consensus Document on Takotsubo Syndrome (Part II): Diagnostic Workup, Outcome, and Management. Eur Heart J. 2018;39:2047–62. 29850820 10.1093/eurheartj/ehy077 PMC5991205

[2] Scally C, Rudd A, Mezincescu A, Wilson H, Srivanasan J, Horgan G, et al. Persistent long-term structural, functional, and metabolic changes after stress-induced (Takotsubo) cardiomyopathy. Circulation. 2018;137:1039–48. 29128863 10.1161/CIRCULATIONAHA.117.031841 PMC5841855

[3] El-Battrawy I, Santoro F, Stiermaier T, Möller C, Guastafierro F, Novo G, et al. Incidence and clinical impact of recurrent Takotsubo syndrome: results from the GEIST registry. J Am Heart Assoc. 2019;8:e010753. 31046506 10.1161/JAHA.118.010753 PMC6512083

[4] Sharkey SW, Windenburg DC, Lesser JR, Maron MS, Hauser RG, Lesser JN, et al. Natural history and expansive clinical profile of stress (tako-tsubo) cardiomyopathy. J Am Coll Cardiol. 2010;55:333–41. 20117439 10.1016/j.jacc.2009.08.057

[5] Tornvall P, Collste O, Ehrenborg E, Järnbert-Petterson H. A case-control study of risk markers and mortality in Takotsubo stress cardiomyopathy. J Am Coll Cardiol. 2016;67:1931–6. 27102508 10.1016/j.jacc.2016.02.029

[6] Templin C, Ghadri JR, Diekmann J, Napp LC, Bataiosu DR, Jaguszewski M, et al. Clinical features and outcomes of Takotsubo (stress) cardiomyopathy. N Engl J Med. 2015;373:929–38. 26332547 10.1056/NEJMoa1406761

[7] Fitzgibbons TP, Edwards YJK, Shaw P, Iskandar A, Ahmed M, Bote J, et al. Activation of inflammatory and pro-thrombotic pathways in acute stress cardiomyopathy. Front Cardiovasc Med. 2017;4:49–9. 28824923 10.3389/fcvm.2017.00049 PMC5541033

[8] Bang DW, Chung JW, Hyon MS, Kim SK, Kwon YJ. Proteomic analysis of serum in patients with apical ballooning syndrome. Int J Cardiol. 2011;146:118–9. 20965584 10.1016/j.ijcard.2010.09.086

[9] Pan X-Y, Zhang Z-W. MFGE8, ALB, APOB, APOE, SAA1, A2M, and C3 as novel biomarkers for stress cardiomyopathy. Cardiovasc Ther. 2020;2020:1615826. 32695227 10.1155/2020/1615826 PMC7350165

[10] Obrutu O, Maughan J, Tjoe B, Herscovici R, Moy P, Rojas N, et al. Smidt Heart Institute Takotsubo Registry – study design and baseline characteristics. American Heart Journal Plus: Cardiology Research and Practice. 2022;13:100086. 38560083 10.1016/j.ahjo.2022.100086 PMC10978169

[11] Ebinger JE, Botwin GJ, Albert CM, Alotaibi M, Arditi M, Berg AH, Binek A, Botting P, Fert-Bober J, Figueiredo JC, Grein JD, Hasan W, Henglin M, Hussain SK, Jain M, Joung S, Karin M, Kim EH, Li D, et al. Seroprevalence of antibodies to SARS-CoV-2 in healthcare workers: a cross-sectional study. BMJ Open. 2021;11:e043584. 33579769 10.1136/bmjopen-2020-043584 PMC7883610

[12] van den Broek I, Fu Q, Kushon S, Kowalski MP, Millis K, Percy A, Holewinski RJ, Venkatraman V, Van Eyk JE. Application of volumetric absorptive microsampling for robust, high-throughput mass spectrometric quantification of circulating protein biomarkers. Clin Mass Spectrom. 2017;4-5:25–33. 39193127 10.1016/j.clinms.2017.08.004 PMC11322776

[13] Fuller G, Njune Mouapi K, Joung S, Shufelt C, van den Broek I, Lopez M, et al. Feasibility of patient-centric remote dried blood sampling: the prediction, risk, and evaluation of major adverse cardiac events (PRE-MACE) study. Biodemogr Soc Biol. 2019;65:313–22. 10.1080/19485565.2020.1765735 PMC8464354 33243027

[14] Mc Ardle A, Binek A, Moradian A, Chazarin Orgel B, Rivas A, Washington KE, et al. Standardized workflow for precise mid- and high-throughput proteomics of blood biofluids. Clin Chem. 2021;68:450–60. 10.1093/clinchem/hvab202 PMC11175165 34687543

[15] Liu Y, Buil A, Collins BC, Gillet LC, Blum LC, Cheng LY, Vitek O, Mouritsen J, Lachance G, Spector TD, Dermitzakis ET, Aebersold R. Quantitative variability of 342 plasma proteins in a human twin population. Mol Syst Biol. 2015;11:786. 25652787 10.15252/msb.20145728 PMC4358658

[16] Teo G, Kim S, Tsou CC, Collins B, Gingras AC, Nesvizhskii AI, et al. MapDIA: preprocessing and statistical analysis of quantitative proteomics data from data independent acquisition mass spectrometry. J Proteomics. 2015;129:108–20. 26381204 10.1016/j.jprot.2015.09.013 PMC4630088

[17] Sundararaman N, Go J, Robinson AE, Mato JM, Lu SC, Van Eyk JE, et al. PINE: an automation tool to extract and visualize protein-centric functional networks. J Am Soc Mass Spectrom. 2020;31:1410–21. 32463229 10.1021/jasms.0c00032 PMC10362945

[18] Bindea G, Mlecnik B, Hackl H, Charoentong P, Tosolini M, Kirilovsky A, et al. ClueGO: a Cytoscape plug-in to decipher functionally grouped gene ontology and pathway annotation networks. Bioinformatics. 2009;25:1091–3. 19237447 10.1093/bioinformatics/btp101 PMC2666812

[19] Ashburner M, Ball CA, Blake JA, Botstein D, Butler H, Cherry JM, et al. Gene ontology: tool for the unification of biology. The Gene Ontology Consortium. Nat Genet. 2000;25:25–9. 10802651 10.1038/75556 PMC3037419

[20] Kanehisa M, Goto S, Kawashima S, Nakaya A. The KEGG databases at GenomeNet. Nucleic Acids Res. 2002;30:42–6. 11752249 10.1093/nar/30.1.42 PMC99091

[21] Gillespie M, Jassal B, Stephan R, Milacic M, Rothfels K, Senff-Ribeiro A, Griss J, Sevilla C, Matthews L, Gong C, Deng C, Varusai T, Ragueneau E, Haider Y, May B, Shamovsky V, Weiser J, Brunson T, Sanati N, et al. The reactome pathway knowledgebase 2022. Nucleic Acids Res. 2021;50:D687–92. 10.1093/nar/gkab1028 PMC8689983 34788843

[22] Pico AR, Kelder T, van Iersel MP, Hanspers K, Conklin BR, Evelo C. Wikipathways: pathway editing for the people. PLoS Biol. 2008;6:e184. 18651794 10.1371/journal.pbio.0060184 PMC2475545

[23] Shimada YJ, Raita Y, Liang LW, Maurer MS, Hasegawa K, Fifer MA, et al. Comprehensive proteomics profiling reveals circulating biomarkers of hypertrophic cardiomyopathy. Circ Heart Fail. 2021;14:e007849. 34192899 10.1161/CIRCHEARTFAILURE.120.007849 PMC8292216

[24] Shi T, Moravec CS, Perez DM. Novel proteins associated with human dilated cardiomyopathy: selective reduction in α(1A)-adrenergic receptors and increased desensitization proteins. J Recept Signal Transduct Res. 2013;33:96–106. 23384050 10.3109/10799893.2013.764897 PMC3624731

[25] Nies MK, Yang J, Griffiths M, Damico R, Zhu J, Vaydia D, et al. Proteomics discovery of pulmonary hypertension biomarkers: Insulin-like growth factor binding proteins are associated with disease severity. Pulm Circ. 2022;12:e12039. 35514776 10.1002/pul2.12039 PMC9063962

[26] Barkovits K, Pacharra S, Pfeiffer K, Steinbach S, Eisenacher M, Marcus K, et al. Reproducibility, specificity and accuracy of relative quantification using spectral library-based data-independent acquisition. Mol Cell Proteomics. 2020;19:181–97. 31699904 10.1074/mcp.RA119.001714 PMC6944235

[27] Molloy MP, Hill C, O’Rourke MB, Chandra J, Steffen P, McKay MJ, et al. Proteomic analysis of whole blood using volumetric absorptive microsampling for precision medicine biomarker studies. J Proteome Res. 2022;21:1196–203. 35166117 10.1021/acs.jproteome.1c00971

[28] Ferreira JP, Verdonschot J, Collier T, Wang P, Pizard A, Bär C, Björkman J, Boccanelli A, Butler J, Clark A, Cleland JG, Delles C, Diez J, Girerd N, González A, Hazebroek M, Huby AC, Jukema W, Latini R, et al. Proteomic Bioprofiles and Mechanistic Pathways of Progression to Heart Failure. Circ Heart Fail. 2019;12:e005897. 31104495 10.1161/CIRCHEARTFAILURE.118.005897 PMC8361846

[29] Egerstedt A, Berntsson J, Smith ML, Gidlöf O, Nilsson R, Benson M, et al. Profiling of the plasma proteome across different stages of human heart failure. Nat Commun. 2019;10:5830. 31862877 10.1038/s41467-019-13306-y PMC6925199

[30] Ho CY, López B, Coelho-Filho OR, Lakdawala NK, Cirino AL, Jarolim P, et al. Myocardial fibrosis as an early manifestation of hypertrophic cardiomyopathy. N Engl J Med. 2010;363:552–63. 20818890 10.1056/NEJMoa1002659 PMC3049917

[31] González A, Schelbert EB, Díez J, Butler J. Myocardial interstitial fibrosis in heart failure. J Am Coll Cardiol. 2018;71:1696–706. 29650126 10.1016/j.jacc.2018.02.021

[32] Bakhshi H, Michelhaugh SA, Bruce SA, Seliger SL, Qian X, Ambale Venkatesh B, et al. Association between proteomic biomarkers and myocardial fibrosis measured by MRI: the multi-ethnic study of atherosclerosis. EBioMedicine. 2023;90:104490. 36857966 10.1016/j.ebiom.2023.104490 PMC10006438

[33] Ansorge HL, Meng X, Zhang G, Veit G, Sun M, Klement JF, et al. Type XIV Collagen Regulates Fibrillogenesis*. J Biol Chem. 2009;284:8427–38. 19136672 10.1074/jbc.M805582200 PMC2659201

[34] Tao G, Levay AK, Peacock JD, Huk DJ, Both SN, Purcell NH, et al. Collagen XIV is important for growth and structural integrity of the myocardium. J Mol Cell Cardiol. 2012;53:626–38. 22906538 10.1016/j.yjmcc.2012.08.002 PMC3472103

[35] Chen S-W, Tung Y-C, Jung S-M, Chu Y, Lin P-J, Kao WWY, et al. Lumican-null mice are susceptible to aging and isoproterenol-induced myocardial fibrosis. Biochem Biophys Res Commun. 2017;482:1304–11. 27939890 10.1016/j.bbrc.2016.12.033

[36] Scally C, Abbas H, Ahearn T, Srinivasan J, Mezincescu A, Rudd A, et al. Myocardial and systemic inflammation in acute stress-induced (Takotsubo) cardiomyopathy. Circulation. 2019;139:1581–92. 30586731 10.1161/CIRCULATIONAHA.118.037975 PMC6438459

[37] Wilson HM, Cheyne L, Brown PAJ, Kerr K, Hannah A, Srinivasan J, et al. Characterization of the myocardial inflammatory response in acute stress-induced (Takotsubo) cardiomyopathy. JACC: Basic to Translational Science. 2018;3:766–78. 30623136 10.1016/j.jacbts.2018.08.006 PMC6314973

[38] Cunin P, Beauvillain C, Miot C, Augusto JF, Preisser L, Blanchard S, Pignon P, Scotet M, Garo E, Fremaux I, Chevailler A, Subra JF, Blanco P, Wilson MR, Jeannin P, Delneste Y. Clusterin facilitates apoptotic cell clearance and prevents apoptotic cell-induced autoimmune responses. Cell Death Dis. 2016;7:e2215–5. 27148688 10.1038/cddis.2016.113 PMC4917652

[39] Krijnen PAJ, Cillessen SAGM, Manoe R, Muller A, Visser CA, Meijer CJLM, et al. Clusterin: a protective mediator for ischemic cardiomyocytes? Am J Phys Heart Circ Phys. 2005;289:H2193-202. 10.1152/ajpheart.00355.2005 15994859

[40] Cubedo J, Padró T, García-Moll X, Pintó X, Cinca J, Badimon L. Proteomic signature of apolipoprotein J in the early phase of new-onset myocardial infarction. J Proteome Res. 2011;10:211–20. 21043527 10.1021/pr100805h

[41] Van Dijk A, Vermond RA, Krijnen PAJ, Juffermans LJM, Hahn NE, Makker SP, et al. Intravenous clusterin administration reduces myocardial infarct size in rats. Eur J Clin Invest. 2010;40:893–902. 20854280 10.1111/j.1365-2362.2010.02345.x

[42] Koller L, Richter B, Winter M-P, Sulzgruber P, Potolidis C, Liebhart F, et al. Clusterin/apolipoprotein J is independently associated with survival in patients with chronic heart failure. J Clin Lipidol. 2017;11:178–84. 28391884 10.1016/j.jacl.2016.11.009

[43] Karnaukhova E. C1-Inhibitor: structure, functional diversity and therapeutic development. Curr Med Chem. 2022;29:467–88. 34348603 10.2174/0929867328666210804085636

[44] Yi X, Jiang D-S, Feng G, J X-j, Zeng H-L. An altered left ventricle protein profile in human ischemic cardiomyopathy revealed in comparative quantitative proteomics. Kardiologia Polska (Polish Heart Journal). 2019;77:951–9. 31434864 10.33963/KP.14936

[45] Rueda F, Borràs E, García-García C, Iborra-Egea O, Revuelta-López E, Harjola VP, et al. Protein-based cardiogenic shock patient classifier. Eur Heart J. 2019;40:2684–94. 31204432 10.1093/eurheartj/ehz294

[46] Ruiz M, Frej C, Holmér A, Guo LJ, Tran S, Dahlbäck B. High-Density Lipoprotein–Associated Apolipoprotein M limits endothelial inflammation by delivering sphingosine-1-phosphate to the sphingosine-1-phosphate receptor 1. Arterioscler Thromb Vasc Biol. 2017;37:118–29. 27879252 10.1161/ATVBAHA.116.308435

[47] Chirinos JA, Zhao L, Jia Y, Frej C, Adamo L, Mann D, Shewale SV, Millar JS, Rader DJ, French B, Brandimarto J, Margulies KB, Parks JS, Wang Z, Seiffert DA, Fang J, Sweitzer N, Chistoffersen C, Dahlbäck B, et al. Reduced Apolipoprotein M and Adverse Outcomes Across the Spectrum of Human Heart Failure. Circulation. 2020;141:1463–76. 32237898 10.1161/CIRCULATIONAHA.119.045323 PMC7200273

[48] Hanff TC, Cohen JB, Zhao L, Javaheri A, Zamani P, Prenner SB, et al. Quantitative proteomic analysis of diabetes mellitus in heart failure with preserved ejection fraction. JACC Basic Transl Sci. 2021;6:89–99. 33665511 10.1016/j.jacbts.2020.11.011 PMC7907637

[49] Martin EA, Prasad A, Rihal CS, Lerman LO, Lerman A. Endothelial function and vascular response to mental stress are impaired in patients with apical ballooning syndrome. J Am Coll Cardiol. 2010;56:1840–6. 21087714 10.1016/j.jacc.2010.03.107 PMC3786427

[50] Barletta G, Del Pace S, Boddi M, Del Bene R, Salvadori C, Bellandi B, et al. Abnormal coronary reserve and left ventricular wall motion during cold pressor test in patients with previous left ventricular ballooning syndrome. Eur Heart J. 2009;30:3007–14. 19700469 10.1093/eurheartj/ehp325

[51] Haines RJ, Pendleton LC, Eichler DC. Argininosuccinate synthase: at the center of arginine metabolism. Int J Biochem Mol Biol. 2011;2:8–23. 21494411 PMC3074183

[52] Surikow SY, Nguyen TH, Stafford I, Chapman M, Chacko S, Singh K, et al. Nitrosative stress as a modulator of inflammatory change in a model of Takotsubo syndrome. JACC: Basic to Translational Science. 2018;3:213–26. 30062207 10.1016/j.jacbts.2017.10.002 PMC6058954

[53] Münzel T, Templin C, Cammann VL, Hahad O. Takotsubo syndrome: impact of endothelial dysfunction and oxidative stress. Free Radic Biol Med. 2021;169:216–23. 33864955 10.1016/j.freeradbiomed.2021.03.033

[54] Zhang Z, Jin S, Teng X, Duan X, Chen Y, Wu Y. Hydrogen sulfide attenuates cardiac injury in takotsubo cardiomyopathy by alleviating oxidative stress. Nitric Oxide. 2017;67:10–25. 28450188 10.1016/j.niox.2017.04.010

[55] Enroth S, Johansson Å, Enroth SB, Gyllensten U. Strong effects of genetic and lifestyle factors on biomarker variation and use of personalized cutoffs. Nat Commun. 2014;5:4684. 25147954 10.1038/ncomms5684 PMC4143927

[56] Enroth S, Bosdotter Enroth S, Johansson Å, Gyllensten U. Effect of genetic and environmental factors on protein biomarkers for common non-communicable disease and use of personally normalized plasma protein profiles (PNPPP). Biomarkers. 2015;20:355–64. 26551787 10.3109/1354750X.2015.1093546

[57] Enroth S, Maturi V, Berggrund M, Enroth SB, Moustakas A, Johansson Å, Gyllensten U. Systemic and specific effects of antihypertensive and lipid-lowering medication on plasma protein biomarkers for cardiovascular diseases. Sci Rep. 2018;8:5531. 29615742 10.1038/s41598-018-23860-y PMC5882890

[58] Michelhaugh SA, Januzzi JL. Finding a needle in a haystack. JACC: Basic to Translational Science. 2020;5:1043–53. 33145466 10.1016/j.jacbts.2020.07.007 PMC7591826

