# Supplementary material for: Takotsubo Syndrome: The First Non-Acute Proteomic Analysis by Remote Dried Blood Microsampling
Source: J Cardiovasc Transl Res. 2026 Feb 27;19(1):32. doi: 10.1007/s12265-026-10752-0 (PMC12948850; doi:10.1007/s12265-026-10752-0)
Supplement: Supplementary file 1 — (DOCX 360 kb) [file 12265_2026_10752_MOESM1_ESM.docx]

**Supplemental Data**


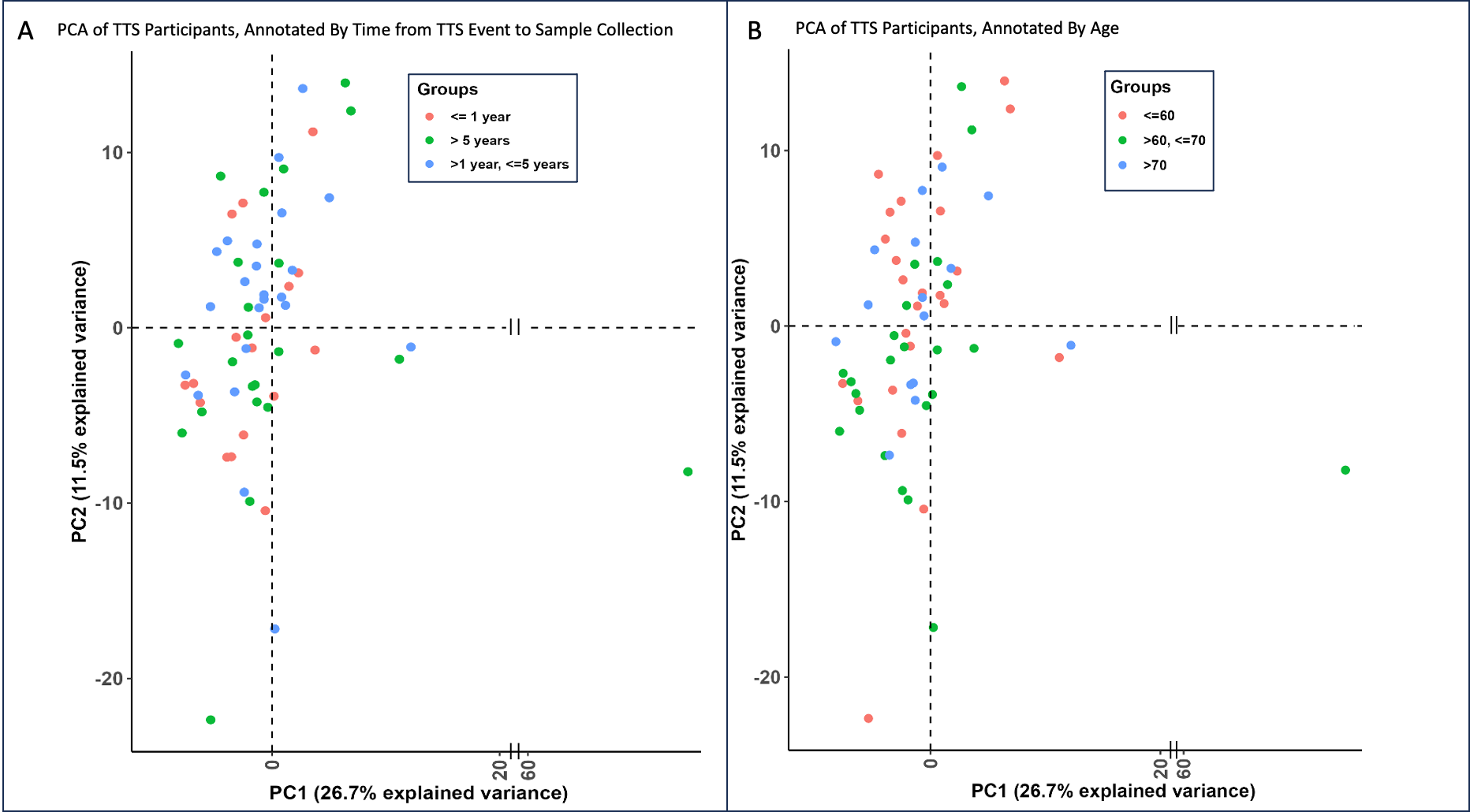


**Supplemental Figure 1. Unsupervised Principal Component Analysis of Takotsubo Participants With Annotation of Time to Sample Collection and Participant Age.** Principal component analysis (PCA) including all 398 quantified proteins in the Takotsubo participants is shown. The samples are annotated based on the time from Takotsubo event to sample collection in (A) and based on the age of the participant at sample collection in (B). There is no distinct clustering in these groups.


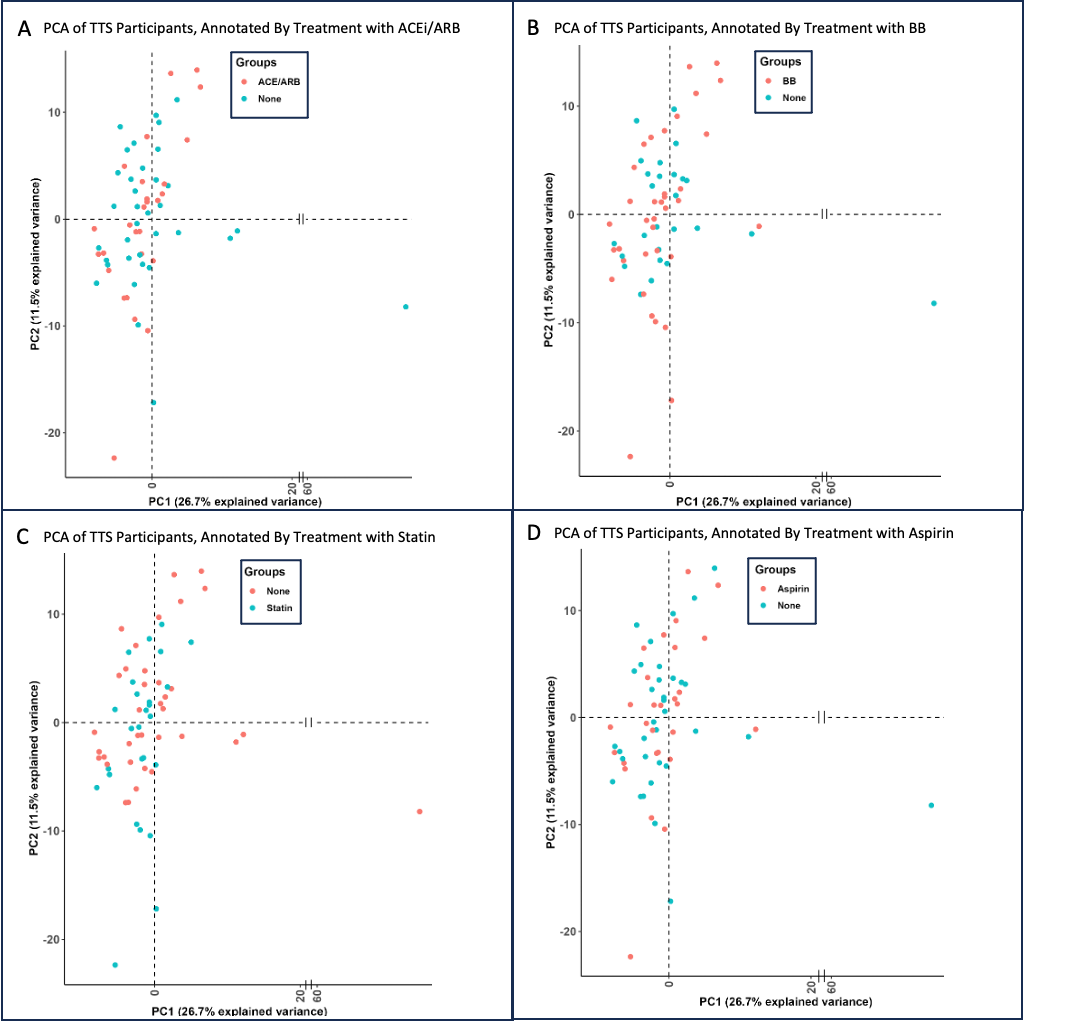


**Supplemental Figure 2.** **Unsupervised Principal Component Analysis of Takotsubo Participants With Annotation of Selected Medications.** A PCA, similar to Supplemental Figure 1, is shown. The samples are annotated based on whether or not participants were treated with angiotensin converting enzyme inhibitors or angiotensin receptor blockers (ACEi/ARBs, panel A), $\beta$-blockers (BB, panel B), HMG-CoA reductase inhibitors (statin, panel C), and aspirin (panel D). There is no clustering based on these groupings.

**Supplemental Table, A-E.** Multivariable linear regression analysis was performed, with the predictors listed below and with the outcome of the protein expression level for each of the top 5 differentially expressed proteins. For each predictor variable, the coefficients with the 95% confidence interval are reported, along with the p-value for the t-statistic in the regression model. Significant p-values at p < 0.05 are bolded. Additional details of the regression model are reported below each table, including the adjusted R^2^ (expressing the amount of variance of protein expression explained by the model) and the F-statistic with its associated p-value (expressing the overall fit of the model).

A. Multivariable linear regression for the outcome of the expression level of Laminin subunit beta-1

|  | **Coefficient**  **(95%** CI**)** | **P-Value** |
| --- | --- | --- |
| **History of Takotsubo Syndrome** | -7767 (-9291, -6244) | **1.55 x 10^-16^** |
| **Age (per 10 years)** | -88 (-158, 18) | **0.01** |
| **Comorbidities** |  |  |
| Diabetes Mellitus | -771 (-3360, 1816) | 0.56 |
| Hypertension | 493 (-986, 1974) | 0.51 |
| Malignancy | 187 (-1671, 2046) | 0.84 |
| **Medications** |  |  |
| ACEi/ARB | -196 (-1865, 1472) | 0.82 |
| $\beta$-blocker | 627 (-943, 2198) | 0.43 |
| Statin | -399 (-1885, 1086) | 0.59 |
| Aspirin | 978 (-785, 2740) | 0.27 |

Note: Adjusted R^2^ = 0.61, F (90) = 18.48, p-value = 4.99 x 10^-17^

B. Multivariable linear regression for the outcome of the expression level of FAM76B

|  | **Coefficient**  (95% CI) | **P-Value** |
| --- | --- | --- |
| **History of Takotsubo Syndrome** | -60187 (-95184, -25189) | **0.001** |
| **Age (per 10 years)** | -1924 (-3536, -311) | **0.02** |
| **Comorbidities** |  |  |
| Diabetes Mellitus | -10878 (-70339, 48581) | 0.72 |
| Hypertension | -2466 (-36474, 31542) | 0.89 |
| Malignancy | 17748 (-24948, 60444) | 0.41 |
| **Medications** |  |  |
| ACEi/ARB | -46985 (-85320, -8650) | **0.02** |
| $\beta$-blocker | 8044 (-28048, 44137) | 0.66 |
| Statin | -22522 (-56646, 11602) | 0.19 |
| Aspirin | 2723 (-37772, 43219) | 0.89 |

Note: Adjusted R^2^ = 0.34, F (90) = 6.74, p-value = 2.45 x 10^-7^

C. Multivariable linear regression for the outcome of the expression level of Chloride intracellular channel protein 4

|  | **Coefficient**  (95% CI) | **P-Value** |
| --- | --- | --- |
| **History of Takotsubo Syndrome** | 73942 (58995, 88889) | **6.51 x 10^-16^** |
| **Age (per 10 years)** | -477 (-1166, 212) | 0.17 |
| **Comorbidities** |  |  |
| Diabetes Mellitus | -7955 (-33351, 17440) | 0.54 |
| Hypertension | 5288 (-9236, 19812) | 0.47 |
| Malignancy | 5475 (-12760, 23710) | 0.55 |
| **Medications** |  |  |
| ACEi/ARB | -4383 (-20756, 11989) | 0.60 |
| $\beta$-blocker | 4589 (-10826, 20004) | 0.56 |
| Statin | 1881 (-12693, 16455) | 0.80 |
| Aspirin | 4810 (-12485, 22106) | 0.58 |

Note: Adjusted R^2^ = 0.57, F (90) = 15.76, p-value = 3.74 x 10^-15^

D. Multivariable linear regression for the outcome of the expression level of Transgelin (22 kDa actin-binding protein)

|  | **Coefficient**  (95% CI) | **P-Value** |
| --- | --- | --- |
| **History of Takotsubo Syndrome** | -3658 (-6480, -835) | **0.01** |
| **Age (per 10 years)** | -38 (-168, 92) | 0.56 |
| **Comorbidities** |  |  |
| Diabetes Mellitus | -1454 (-6249, 3340) | 0.55 |
| Hypertension | 2250 (-492, 4992) | 0.11 |
| Malignancy | -212 (-3655, 3231) | 0.90 |
| **Medications** |  |  |
| ACEi/ARB | -613 (-3705, 2478) | 0.69 |
| $\beta$-blocker | -1049 (-3959, 1862) | 0.48 |
| Statin | -1127 (-3879, 1624) | 0.42 |
| Aspirin | 1351 (-1913, 4617) | 0.41 |

Note: Adjusted R^2^ = 0.08, F (90) = 1.90, p-value = 0.06

E. Multivariable linear regression for the outcome of the expression level of Collagen alpha-1(XIV) chain

|  | **Coefficient**  (95% CI) | **P-Value** |
| --- | --- | --- |
| **History of Takotsubo Syndrome** | -4999 (-6913, -3086) | **1.29 x 10^-6^** |
| **Age (per 10 years)** | -89 (-177, -1) | **0.05** |
| **Comorbidities** |  |  |
| Diabetes Mellitus | 1748 (-1503, 4999) | 0.29 |
| Hypertension | -228 (-2088, 1631) | 0.81 |
| Malignancy | -1152 (-3486, 1182) | 0.33 |
| **Medications** |  |  |
| ACEi/ARB | -518 (-2613, 1578) | 0.62 |
| $\beta$-blocker | -362 (-2335, 1611) | 0.72 |
| Statin | -14 (-1880, 1851) | 0.99 |
| Aspirin | -519 (-2733, 1695) | 0.64 |

Note: Adjusted R^2^ = 0.36, F (90) = 7.43, p-value = 4.80 x 10^-8^
